# Physiological Optimization of Digital Self-Disimpaction Using a Step Stool: A Case Report

**DOI:** 10.7759/cureus.114496

**Published:** 2026-08-13

**Authors:** Munehiro Yoshitomi

**Affiliations:** 1 Department of Emergency Medicine, Emergency Center, St. Mary's Hospital, Kurume, JPN

**Keywords:** anorectal angle, case report, digital disimpaction, fecal impaction, squatting position, vasovagal reflex

## Abstract

Digital disimpaction, which is often required for cases of severe fecal impaction, carries significant risks of mucosal injury and vasovagal reflex. Evidence-based guidance for safe home-care disimpaction is limited. Moreover, detailed physiological self-assessments of medically trained individuals during such events have seldom been reported. Herein, we report a rare case of self-disimpaction performed by a gastroenterological surgeon, providing a detailed clinical analysis of the physiological mechanisms involved. A 49-year-old male gastroenterologist presented with recurrent distal fecal impaction associated with occupational constipation. Previous evacuation attempts in a sitting posture required excessive strain and triggered severe presyncopal symptoms (bradycardia and diaphoresis). In the current episode, initial sitting attempts reproduced these vagal prodromes. Deep hip flexion was achieved using a toddler’s step stool (height: approximately 20 cm) to simulate a squatting posture. This positional change immediately relieved mechanical obstruction and autonomic symptoms. By maintaining rigorous surgical nail hygiene, the patient successfully fragmented and evacuated the stool without hemorrhage or mucosal trauma. Footstool-assisted squatting induces puborectalis muscle relaxation and straightens the anorectal angle. This biomechanical realignment reduces outlet resistance and minimizes the requirement for forceful straining, thereby preventing hemodynamic collapse secondary to vagal stimulation. Simulating physiological squatting posture and ensuring strict nail hygiene may be practical strategies for mitigating hemodynamic instability and mechanical injury during digital disimpaction. Importantly, these findings were derived from a single healthy adult. Thus, this technique cannot be generalized to high-risk populations (e.g., the elderly or those with cardiac disease) without professional medical supervision.

## Introduction

Fecal impaction is a common and potentially severe complication of chronic constipation that predominantly affects the elderly and institutionalized populations. It significantly diminishes the quality of life and can lead to stercoral ulceration, obstruction, and perforation. When a hard stool impacts the distal rectum, manual or digital disimpaction is often necessary for rescue intervention, despite its invasiveness [1].

Despite its necessity and frequency in home care settings, digital disimpaction is not a benign procedure. It carries distinct iatrogenic risks, primarily mechanical mucosal trauma such as rectal bleeding [2]. Even more critical are the systemic hemodynamic risks; forceful rectal manipulation and excessive straining can trigger profound vagal responses, with many prior reports of bradycardic arrest and "death by disimpaction" [3]. However, there is a notable paucity of formalized, evidence-based guidelines for patient-initiated or caregiver-assisted techniques that specifically address optimal anatomical positioning and risk mitigation for this procedure. Crucially, although the existing literature describes generalized risks, there is a distinct dearth of detailed, first-hand accounts from medically trained individuals capable of conducting precise physiological self-assessment during such acute events. This case report provides important first-person clinical observations of successful self-disimpaction by a medically trained individual, providing detailed insights into the immediate physiological and autonomic responses to anatomical positioning and professional hygiene. This study followed the CARE guidelines [4]. This report is intended strictly as a physiological and mechanistic observation to inform future clinical guidance, rather than a direct how-to guide for unsupervised lay self-treatment.

## Case presentation

Patient profile and history 

The patient was a 49-year-old male gastroenterological surgeon without a significant medical history of diabetes mellitus, cardiovascular disease, or structural colorectal abnormalities. Routine colonoscopic surveillance, most recently performed six months prior, was unremarkable. However, the patient reported a long-standing history of severe constipation induced by the professional environment. The patient’s irregular bowel habits were exacerbated by prolonged working hours, inconsistent hydration, and occasional self-directed use of over-the-counter loperamide during periods of demanding clinical duties. These habits collectively contributed to the development of chronic constipation, leading to fecal retention and hardening, resulting in infrequent bowel movements (1-2 per week).

Episode 1 (prior event) 

At the age of 46 years, the patient experienced severe distal fecal impaction. He attempted self-evacuation in a conventional sitting position on a standard Western-style toilet. The stool was rock-hard, necessitating excessive and forceful straining (Valsalva maneuver) against the closed anal canal. This effort provoked severe anal pain and rapid onset of profound vasovagal symptoms, including marked diaphoresis, nausea, and an intense subjective sensation of bradycardia and presyncope. Fearing imminent loss of consciousness, he immediately sought urgent gastroenterology care, where he underwent professional digital disimpaction. These intense subjective sensations of bradycardia and presyncope were self-reported without objective hemodynamic monitoring (e.g., electrocardiography or blood pressure measurement).

Episode 2 (current event) 

**Figure 1:**
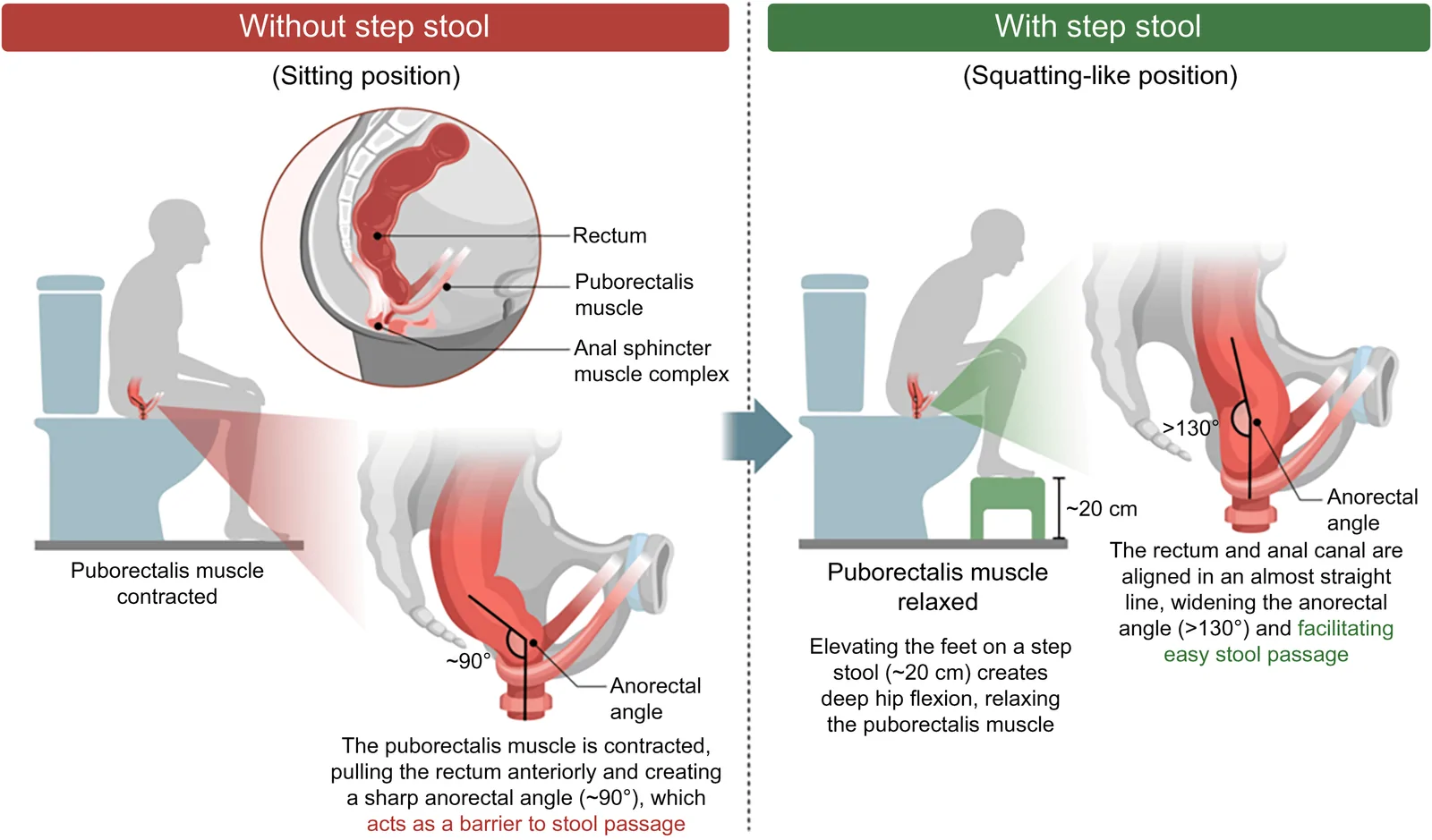
Overview of the patient’s biomechanical realignment of the anorectal angle using a step stool (A) The conventional sitting posture maintains puborectalis contraction and a sharp anorectal angle. (B) Deep hip flexion using a step stool relaxes the muscle and straightens the anorectal passage. Note: The anorectal angle values (approximately 90° and >130°) are literature-derived reference ranges used to illustrate the general anatomical mechanism, not empirical measurements obtained from this specific patient. Furthermore, these principles of postural mechanics, originally established for spontaneous defecation, are conceptually extrapolated here to the distinct scenario of digital disimpaction. Image credit: This original illustration was created by professional medical illustrators at Editage.

Three years later, at the age of 49 years, the patient (note: the patient in this case is the author of this report) experienced recurrence of fecal impaction after three days without bowel movement. Digital self-examination revealed a large rock-hard fecal mass that had impacted immediately above the anal verge. Initial attempts at self-disimpaction in a conventional sitting position triggered rapid vagal prodromes (nausea, diaphoresis, and lightheadedness) within a few minutes. Recognizing the physiological disadvantages and impending syncope, the patient acquired a toddler's step stool (height, approximately 20 cm), which was placed in front of the toilet. Placing both feet on the stool elevated his knees significantly above his hips, creating deep hip flexion and simulating a "squatting" posture (Figure 1). The consequent physiological effects were rapid; the sensation of mechanical obstruction at the anorectal junction diminished, and autonomic prodromes subsided within approximately 1-2 min as the requirement for excessive straining was alleviated, although these responses were also self-reported and without objective monitoring. The patient meticulously maintained short, trimmed, and smooth fingernails per standard surgical hygiene, applied lubrication, and was able to insert his index finger to hook and gently fragment the fecal mass. Aided by the improved anatomical angle, the feces were evacuated smoothly. The procedure was completed without any hemorrhage (confirmed by visual inspection of the stool and toilet tissue), mucosal injury, or residual pain.

Follow-up and outcome 

Following successful self-disimpaction, the patient experienced immediate relief. No subsequent medical interventions were required. To prevent recurrence, the patient subsequently adopted a daily regimen comprising increased fluid intake, dietary modifications, and regular use of step stools during defecation. No recurrence of fecal impaction was observed during follow-up.

## Discussion

Principal finding

This case, which involved the self-analysis by a specialist surgeon, highlights two critical pathophysiological mechanisms governing the safety of digital disimpaction: the biomechanics of the anorectal angle and hemodynamics of the Valsalva maneuver.

Biomechanical advantage of the squatting position 

The primary factor determining the success and safety of Episode 2 was the alteration of the anorectal angle. Historically, according to Tagart, in a conventional sitting position, the puborectalis sling remains contracted, pulling the distal rectum anteriorly to create a sharp "kink" of approximately 90°. This angulation functions as a physiological valve for continence, but presents a significant physical barrier during the evacuation of hard stools [5]. Subsequent clinical and radiographic studies by Sikirov demonstrated that a squatting posture induces essential muscular relaxation, straightening the anorectal angle to >130°, and significantly reducing the need for straining [6].

Recently, a prospective clinical trial by Modi et al. robustly confirmed that the implementation of a defecation posture modification device (a footstool) safely and effectively minimizes bowel movement duration and expulsion effort in a cohort of healthy individuals [7]. Furthermore, contemporary clinical guidelines published by the Italian Unitary Society of Colon-Proctology explicitly advocated for such postural modifications as a fundamental first-line behavioral measure for patients presenting with obstructed defecation patterns [8].

Crucially, contemporary magnetic resonance imaging studies by Kıraç et al. quantitatively reinforced these concepts, confirming that widening of the anorectal angle and relaxation of the puborectalis muscle are fundamental for effective defecation [9]. These radiographic findings are strongly supported by well-indexed magnetic resonance defecography studies, such as those by Thapar et al., which visually and quantitatively demonstrated dynamic normalization and straightening of the anorectal junction occurring during simulated evacuation [10]. In the present case, deep hip flexion afforded by the footstool facilitated biomechanical realignment, aligning the longitudinal axes of the rectum and anal canal, thereby minimizing outlet resistance and permitting digital access with minimal effort.

Hemodynamic stability and autonomic response 

In Episode 1, the sharp anorectal angle necessitated a forceful Valsalva maneuver. According to a review by Kapoor, a prolonged Valsalva maneuver significantly increases intrathoracic pressure, which impedes venous return to the heart (decreased preload), culminating in a drop in cardiac output, consequently precipitating syncope [11]. Furthermore, forceful rectal manipulation can trigger a vagally mediated bradycardic reflex. In Episode 2, straightening of the anorectal passage via the footstool dramatically reduced the need for straining. Additionally, the simulated squatting position involved mechanical compression of the lower limbs and abdomen, which aided venous return and counteracted the hypotension associated with vagal stimulation.

Clinical implications and "surgeon's hygiene"

The risk of traumatic laceration during blind digital manipulation is highly critical. The author’s professional habit of rigorous nail hygiene served as a fundamental preventive measure in this case. From an occupational health perspective, self-directed use of loperamide and intentional bowel suppression during demanding clinical duties represent significant, modifiable behavioral risk factors that are common among physicians. Furthermore, it is crucial to note the significant discrepancy between the high-risk demographic typically affected by fecal impaction (elderly and institutionalized patients) and the healthy profile of the current case. While previous studies have validated the mechanical benefits of the squatting posture for spontaneous defecation, its safety profile during instrumented digital fragmentation has not been formally evaluated. Therefore, lay caregivers should be instructed to seek professional medical evaluation rather than attempt digital disimpaction independently. The "surgeon's hygiene" concept should only be applied under explicit clinical guidance. Ultimately, if vagal prodromal symptoms occur during any attempt, the absolute safest course of action is to immediately cease the procedure and seek urgent medical care.

Limitations 

This report describes a single case involving a medically trained individual, which inherently limits the generalizability of the technical execution of this approach. Furthermore, the physiological observations were based on subjective self-reporting and without any objective hemodynamic monitoring during the acute phase. Moreover, the causal attribution of symptom relief to the postural change is limited by the single within-subject design and lacks objective hemodynamic monitoring or a control condition. Consequently, this report should not be interpreted as an encouragement for lay self-disimpaction, particularly in elderly or medically complex patients, who must seek professional evaluation.

## Conclusions

Overall, this single-case observation demonstrates a plausible physiological mechanism where a footstool-assisted squatting posture may straighten the anorectal angle and reduce the expulsive effort required during digital disimpaction. However, these hypothesis-generating findings derive from a low-risk individual, warranting formal validation before informing general clinical practice. Individuals experiencing fecal impaction, particularly the elderly or medically complex patients, should be strongly encouraged to seek prompt medical evaluation rather than attempt self-disimpaction. Finally, if vagal prodromal symptoms occur during any self-management attempt, immediately stopping the procedure and seeking urgent care are imperative, regardless of postural modification.
